# Different LED Light Wavelengths and Photosynthetic Photon Flux Density Effect on *Colletotrichum acutatum* Growth

**DOI:** 10.3390/plants11010143

**Published:** 2022-01-05

**Authors:** Neringa Rasiukevičiūtė, Aušra Brazaitytė, Viktorija Vaštakaitė-Kairienė, Alma Valiuškaitė

**Affiliations:** 1 Laboratory of Plant Protection, Institute of Horticulture, Lithuanian Research Centre for Agriculture and Forestry, Kauno Street 30, LT-54333 Babtai, Lithuania; alma.valiuskaite@lammc.lt; 2 Laboratory of Plant Physiology, Institute of Horticulture, Lithuanian Research Centre for Agriculture and Forestry, Kauno Street 30, LT-54333 Babtai, Lithuania; ausra.brazaityte@lammc.lt (A.B.); viktorija.vastakaite-kairiene@lammc.lt (V.V.-K.)

**Keywords:** conidia, growth, mycelium, PFD

## Abstract

The study aimed to evaluate the effect of different photon flux density (PFD) and light-emitting diodes (LED) wavelengths on strawberry *Colletotrichum acutatum* growth characteristics. The *C. acutatum* growth characteristics under the blue 450 nm (B), green 530 nm (G), red 660 nm (R), far-red 735 nm (FR), and white 5700 K (W) LEDs at PFD 50, 100 and 200 μmol m^−^^2^ s^−1^ were evaluated. The effect on *C. acutatum* mycelial growth evaluated by daily measuring until five days after inoculation (DAI). The presence of conidia and size (width and length) evaluated after 5 DAI. The results showed that the highest inhibition of fungus growth was achieved after 1 DAI under B and G at 50 μmol m^−2^ s^−1^ PFD. Additionally, after 1–4 DAI under B at 200 μmol m^−2^ s^−1^ PFD. The lowest conidia width was under FR at 50 μmol m^−2^ s^−1^ PFD and length under FR at 100 μmol m^−2^ s^−1^ PFD. Various LED light wavelengths influenced differences in *C. acutatum* colonies color. In conclusion, different photosynthetic photon flux densities and wavelengths influence *C. acutatum* growth characteristics. The changes in *C. acutatum* morphological and phenotypical characteristics could be related to its ability to spread and infect plant tissues. This study’s findings could potentially help to manage *C. acutatum* by LEDs in controlled environment conditions.

## 1. Introduction

Nowadays, one of the serious problems is food production contamination by various sources as chemical pesticides, phytotoxins, chemicals for food processing, and others [1]. In food production, the residues of chemical pesticides are present as contaminants in higher than safe concentrations [2,3,4]. However, traditionally, fungicides are used for disease control by routine application [5,6]. Besides this, chemical pesticides negatively affect beneficial organisms in the environment, pollute water, food, soil, and affect animal and human health due to frequent use [2,3,7,8,9]. Furthermore, the growing demand for safe food leads to alternative plant protection because chemical pesticides have side effects and induce resistance to pathogens [10,11]. Additionally, the European Union (EU) directive 2009/128/EC and Green Deal promotes sustainable pesticides use. Therefore, various physical, biological, and integrated plant protection strategies are applied to prolong and maintain the shelf-life of horticultural crops [12,13,14,15]. Furthermore, plant protection should be based on new substances and technologies for integrated harmful organism control with the least harmful methods for humans and the environment [3,9,12,14,15].

The *Colletotrichum* genus pathogens complex is one of the primary disease-causing strawberry fruit contaminations. Strawberry anthracnose (*Colletotrichum* spp.) can infect up to 80% of nurseries plants and reduce yield by over 50%. The *Colletotrichum* spp. (*C. acutatum*, *C. fragariae*, and *C. gloeosporioides*) infects a wide diversity of hosts and is an important horticultural crop in southern countries. The optimal temperature for pathogen development is between +26.7 and 32 °C, but it adapts to cooler climatic conditions concerning warm day periods regarding climate change [12,16,17,18,19,20]. The *Colletotrichum* species complex comprises about 190 species, of which three species, *C. acutatum*, *C. gloeosporioides*, and *C. fragariae*, cause strawberry anthracnose [19,20]. The microbial contamination of fruits reduces their shelf-life and occurs during harvesting and postharvest [21].

Light is essential for all crops, as it induces photosynthesis, which is crucial for plant growth and development. In addition, in greenhouses and closed environmental conditions, supplemental light eliminates cropping season and improves nutritional quality, decreases nitrate concentration, increases yield quality, and influences plants’ grown parameters. The role of light on various crops was evaluated [22,23,24,25]. However, fungal pathogens also react to solar radiation, and it depends on the duration of exposure, wavelengths, and irradiance [26]. Fungi sense light using 11 photoreceptors, which control morphological and physiological responses [27]. Sunlight induces photomorphogenesis, phototropism and serves for signal orientation [28]. The ultraviolet B radiation (UV-B) could positively or negatively affect fungal growth and conidia germination [26]. Fungal responses to light are reflected by conidia production, stress tolerance, pigmentation, virulence, second metabolism, and germination speed [29]. Therefore, LED light use for plant pathogens control relies on sustainable technologies for integrated harmful organism control [13,14,15]. It has been reported that blue LED light could suppress the sporulation and germination of *Penicillium* spp., *Phomopsis* spp., *Botrytis* spp., and *Aspergillus* spp. Additionally, blue, red, and far-red inhibit *Aspergillus* spp. and other pathogens [8,9,30,31]. Additionally, blue, green, and red LED lights can induce systemic resistance to pathogens [14]. However, the light exposure time should be economically efficient [32]. In addition, 50–150 μmol m^−2^ s^−1^ PFD of blue LED induced *B. cinerea* resistance in tomatoes [8].

It was previously reported that LED light affects pathogens. However, a lack of different photosynthetic photon flux densities and different wavelengths impact *C. acutatum*. In addition, *C. acutatum* morphological and phenotypical characteristics could be related to its ability to spread and infect plant tissues. Therefore, we assume that different photon flux densities and wavelengths could suppress or stimulate plant pathogens. Furthermore, not all studies evaluated morphological and phenotypical characteristics of *C. acutatum* that could influence pathogen ability to infect plant tissues and spread. Therefore, the present study aimed to evaluate the effect of different photon flux densities and LED light wavelengths on strawberry *C. acutatum* growth characteristics in vitro.

## 2. Results

**Figure 1:**
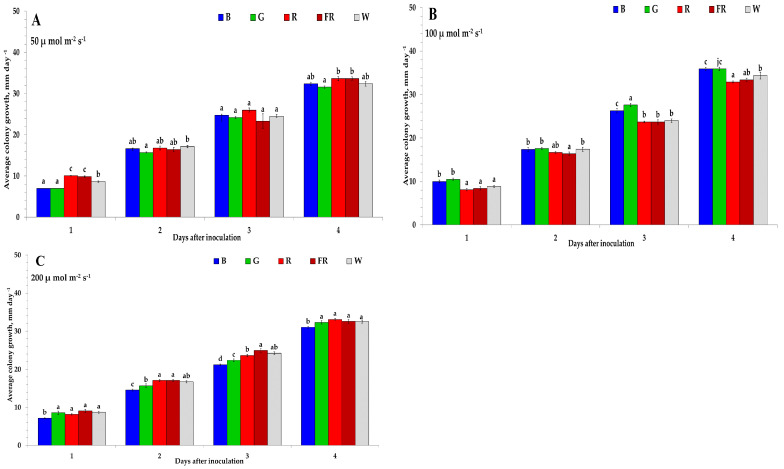
The average *C. acutatum* mycelium growth rate under various photosynthetic photon flux densities and LED light wavelength. (**A**)—50 μmol m^−2^ s^−1^, (**B**)—100 μmol m^−2^ s^−1^, (**C**)—200 μmol m^−2^ s^−1^. B—blue (peak = 450 nm); G—green (peak = 530 nm); R—red (peak = 660 nm); FR—far red (peak = 735 nm); W—white (5700 K). The values in the figure are expressed as mean ± standard error (*n* = 4). According to Duncan’s multiple range test, the means of different letters are significantly different at the *p* < 0.05 level.

The *C. acutatum* was exposed under different LEDs wavelengths, and PFD revealed morphological and phenotypic characteristics difference. In order to evaluate the intensity of LED light effect on *C. acutatum* growth characteristics, the isolates exposed B, G, R, FR, and W and 50, 100 and 200 μmol m^−2^ s^−1^ PFD. We observed that *C. acutatum* acted differently under different PFD and LEDs. In addition, the mycelium growth was different for each PFD. The LEDs influenced *C. acutatum* mycelium growth, acting differently under different light conditions. The lowest *C. acutatum* mycelium growth at 50 μmol m^−2^ s^−1^ PFD observed after 1 DAI was under B and G, at 2 DAI—G, at 3 DAI—FR, and 4 DAI—G (Figure 1A). In addition, the highest mycelium growth rate was under R at 1–4 DAI, besides at 1 and 4 DAI under FR. The MGI inhibition of *C. acutatum* mycelium growth under different wavelengths and 50 μmol m^−2^ s^−1^ PFD, at 2 DPI reached—0.95% G and 4 DPI 1.45%.

The results showed that at 100 μmol m^−2^ s^−1^ PFD, the highest inhibition of *C. acutatum* mycelium growth was under R after 1 and 3–4 DAI (Figure 1B). Additionally, the after 2–3 DAI FR compared with other treatments. However, G increased the mycelium growth at 1–4 DAI and B at 1 and 4 DAI. The MGI inhibition at 2 DAI reached 2.38% under FR and at 4 DAI—2.66% under R.

However, the highest inhibition of *C. acutatum* mycelium growth was observed at 200 μmol m^−2^ s^−1^ PFD under B after 1–4 DAI (Figure 1C). Besides, the highest mycelium growth was observed under FR at 1–3 DAI and R at 2 and 4 DAI. The MGI inhibition rate at 2 DAI—12.33% and 4 DAI—4.69% under B.

Different wavelengths influenced the mycelial growth of the *C. acutatum* at various PFD. Overall, our data show that the highest inhibition of *C. acutatum* after 4 DAI was at 50 μmol m^−2^ s^−1^ PFD under G, at 100 μmol m^−2^ s^−1^ PFD—R, and 200 μmol m^−2^ s^−1^ PFD under B.

**Figure 2:**
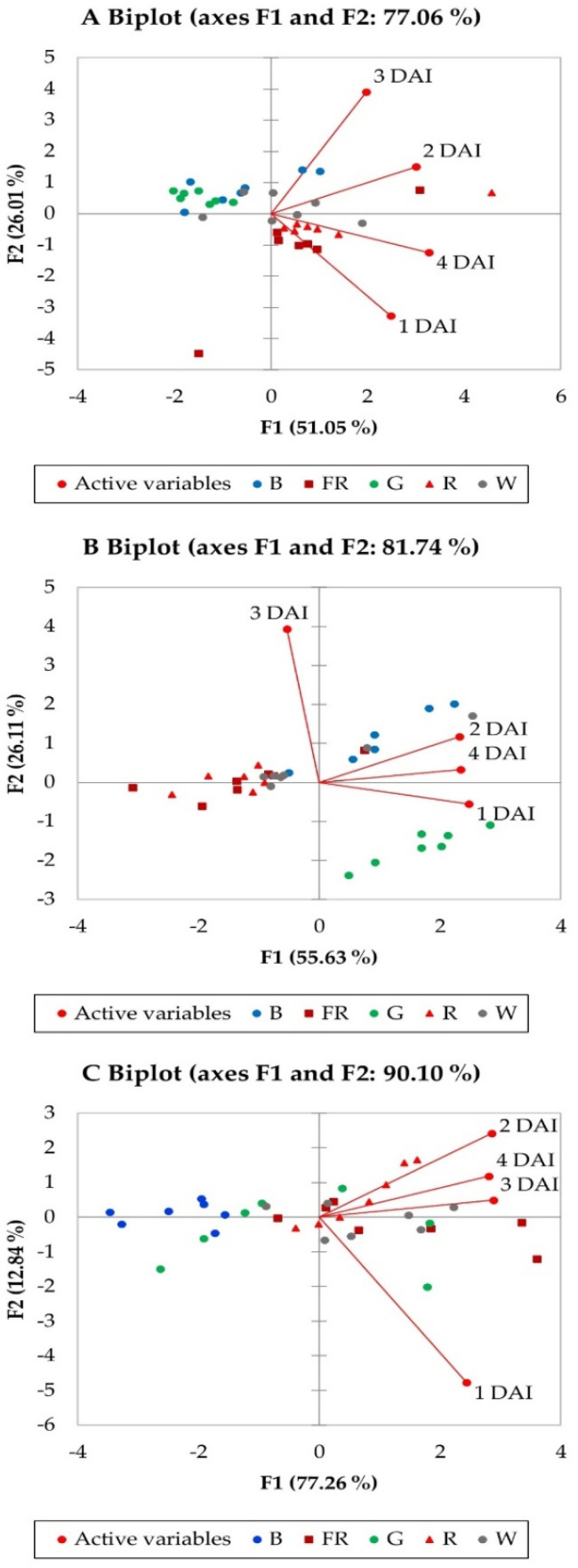
A principal component analysis biplot, indicating distinct effects of lighting treatments on *C. acutatum* mycelium growth rate under various photosynthetic photon flux densities and LED light wavelength. (**A**)—50 μmol m^−2^ s^−1^, (**B**)—200 μmol m^−2^ s^−1^, (**C**)—200 μmol m^−2^ s^−1^. B—blue (peak = 450 nm); G—green (peak = 530 nm); R—red (peak = 660 nm); FR—far red (peak = 735 nm); W—white (5700 K). DAI—days after inoculation.

The PCA biplots show relationships between the average measurements of *C. acutatum* mycelium growth on 1–4 DAI under the lighting of B, G, R, FR, W at 50 (Figure 2A), 100 (Figure 2B), and 200 µ mol m^−2^ s^−1^ (Figure 2C). The PCA biplots generally showed distinct effects of B and G to R and FR regardless of PFD. The PCA factor loadings, scores, and eigenvalues for the first two principal components (F1 and F2) are presented in Table 1. The first two PCAs extracted from the components amounted to 77.06% of the total data variance for 50 µ mol m^−2^ s^−1^, 81.74% for 100 µ mol m^−2^ s^−1^, and 90.10% for 100 µ mol m^−2^ s^−1^ PFDs. In PCA biplots (Figure 2A–C), two vectors with an angle <90° show a positive correlation, and two vectors with an angle >90° have a negative correlation. At 50 µ mol m^−2^ s^−1^, moderate significant positive correlations between 1 DAI, 2 DAI, and 4 DAI, and weak significant positive correlation between 2 DAI and 3 DAI (Table S1). At 100 µ mol m^−2^ s^−1^ PFD, a significantly strong correlation between 1 DAI and 2 DAI was determined, and a significant moderate correlation between 2 DAI and 4 DAI (Table S2). At 200 µ mol m^−2^ s^−1^ PFD, a significant very strong correlation between 2 DAI and 3 DAI and 4 DAI, a significantly strong correlation between 3 DAI and 4 DAI, and 1 DAI with 3 DAI, as well as a significant moderate correlation between 1 DAI and 2 DAI, was observed (Table S3).

To evaluate the associations between DAI’s and lighting treatments on *C. acutatum* mycelium growth, the PCA biplots were analyzed according to F1 and F2 factor loadings and scores (Table 1; Figure 2A–C). At 50 µ mol m^−2^ s^−1^ PFD, the associations between 1 DAI and 4 DAI to R and FR lighting treatments were found. In addition, 2 DAI and 3 DAI were associated with W lighting. None of the DAI’s was associated with B and G treatments (Figure 2A). At 100 µ mol m^−2^ s^−1^ PFD, 1 DAI was associated with G, 2 DAI and 4 DAI to B, and 3 DAI to R, FR, and W lighting treatments (Figure 2B). At 200 µ mol m^−2^ s^−1^ PFD, 1 DAI was associated with FR and W, and 2 DAI, 3 DAI, and 4 DAI to R treatment. It was found that B and G lighting treatments were not associated with *C. acutatum* mycelium growth at all investigated DAIs at 200 µ mol m^−2^ s^−1^ PFD (Figure 2C).

The principal component analysis demonstrated distinct effects of lighting treatments at different PFD levels (50, 100 and 200 µ mol m^−2^ s^−1^) on *C. acutatum* mycelium growth at all investigated days after infection (1 to 4 DAI). However, similar trends were observed between associations of 1 DAI and 4 DAI to R and FR lighting at 50 and 200 µ mol m^−2^ s^−1^ PFDs. Contrary to this, at 100 µ mol m^−2^ s^−1^ PFD, such lighting treatments were associated with 3 DAI.

Different wavelengths influenced the mycelial growth curve (AUMGC) of the *C. acutatum* at various PFD. The mycelial growth curve (AUMGC) was affected by PFD and different wavelengths (Table 2). The mycelial growth curve (AUMGC) of the *C. acutatum* slightly was reduced by G (9.94%) at 50 μmol m^−2^ s^−1^ PFD and 200 μmol m^−2^ s^−1^ PFD under B (9.37%), besides at 100 μmol m^−2^ s^−1^ under R (10.30%) compared with other treatments.

The conidia size (width and length) characteristics of *C. acutatum* isolates were studied under five different LEDs wavelengths and three PFD (Table 3). The results revealed that conidial dimensions differed among isolates under the different LEDs and PFD, ranging from 7.6 to 10.3 μm in width and 21.5 to 33.3 μm in length. Our results showed that at 50 μmol m^−2^ s^−1^ PFD, our conidia size varied from 7.6 to 10.3 μm in width and from 23.8 to 33.3 μm in length. The lowest conidia width was under FR and length under G. The highest width of conidia was under W at 50 μmol m^−2^ s^−1^ PFD. The conidia length was smallest under FR and width under G at 100 μmol m^−2^ s^−1^ PFD. However, the highest conidia size in width and length were under W. The dimensions of conidia at 200 μmol m^−2^ s^−1^ PFD ranged from 8.3 to 9.4 μm in width and from 24.9 to 30.5 μm in length. The lowest conidia width was under G and length under R. The diameter of conidia was the largest, 9.4 μm at B in width and 30.5 μm at FR in length.

**Figure 3:**
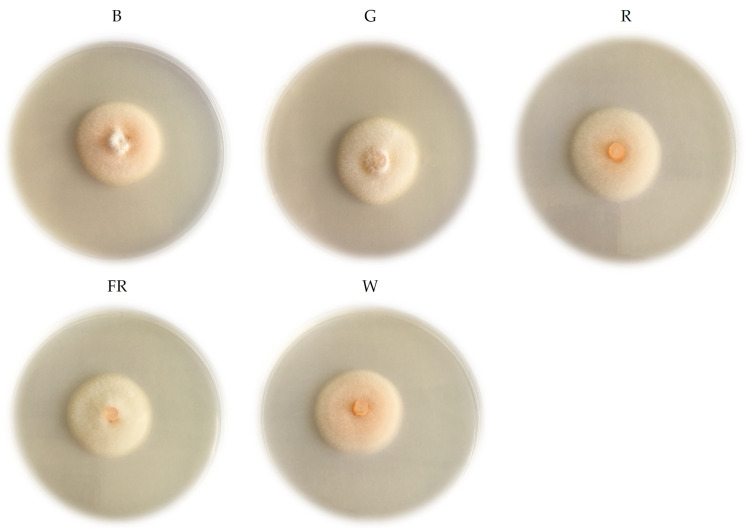
The mycelium appearance of *C. acutatum* under various LED light wavelengths. B—blue (peak = 450 nm); G—green (peak = 530 nm); R—red (peak = 660 nm); FR—far red (peak = 735 nm); W—white (5700 K).

The color of *C. acutatum* upper mycelium under different wavelengths varied irrespectively to PFD (Figure 3). The *C. acutatum* colonies could be grouped into three types based on the basis observed in color pigments. The same color tendency was under all three evaluated PFD. The color ranged from white to orange. The *C. acutatum* mycelium color under G, R and FR was light orange, and under B and W—orange color at 5 DAI after incubation under different wavelengths. Overall, *C. acutatum* colonies’ color varied among different LED light wavelengths.

To determine the recovery of the *C. acutatum* after different LEDs and PFD influence, the re-isolation was after 5 DAI (Table 4). *C. acutatum* colonies, after re-isolation, visually looked similar to the original isolates. 

However, the data revealed that recovery was slower at 50 μmol m^−2^ s^−1^ PFD under B than other wavelengths. Additionally, under R recovered faster than under other LEDs. However, at 100 μmol m^−2^ s^−1^ PFD, the slowest recovery was observed under W and faster under G. Besides, at 200 μmol m^−2^ s^−1^ PFD in B recovered slowest, and under W recovered faster than under other wavelengths.

## 3. Discussion

The LEDs could be an innovative tool for environmentally friendly pathogen control in controlled environmental conditions. The LEDs allow a selection of a specific light spectrum and PFD to prevent horticultural crops damaged by pathogens. However, literature research showed a lack of studies on the light-mediated effect on specific wavelength and PFD, but not as in our study [29,30,33,34,35]. Fungi realize red, green, near-ultraviolet, blue, and far-red with 11 photoreceptors. Mostly all fungi have two types of photoreceptors, while some even have three. In addition, light for fungi may give an orientation for reproduction and growth [27,28].

Fungi use light combined with the circadian clock to adapt to stress and reproductive structures productions at precise time and place [36]. This study observed that *C. acutatum* acted differently under various LEDs and PFD. Different LEDs and PFD could suppress or stimulate plant pathogens as fungi respond to light [8,9,14,27,28,35]. Therefore, the LEDs and PFD inhibitory effect could be related to the pathogen’s ability to infect plant tissues and spread. As light influences plant growth and development for all crops, selecting specific spectra eliminates cropping season in closed environment conditions and influences plants’ grown parameters and nutritional quality [22,23,24,25]. Therefore, a knowledge of LEDs and PDF effect on *C. acutatum* is essential for combined plants cultivation in closed environmental conditions. The present study focused on the various LEDs and PFD effects on *C. acutatum* in vitro.

Recently, several reports had a light effect on the development, growth of fungi, and inhibition. Most studies agree that light influences various fungal pathogens, not many on *C. acutatum.* For example, it’s reported that light quality influences rose *Podosphaera pannosa* development and growth [35]. The continuous blue light (40 μmol m^−^^2^ s^−^^1^) or dark-light regime reduced the mycelium growth of *Penicillium italicum* and *P. citri* [30]. In addition, *B. cinerea* illumination by purple light (400–410 nm) and blue (450–460 nm at 12 h photoperiod inhibited mycelium growth [33]. Besides this, *C. capsici* lowest growth was under 24 h dark photoperiod [37]. In addition, *C. gloeosporioides* irradiation by continuous 25 mW/cm^2^ of 405 nm light for 40 min causes morphological changes in its colony [38]. These findings indicate that various fungi mycelium growth was affected under different light. Our results showed the highest inhibition of *C. acutatum* after 4 DAI at 50 μmol m^−2^ s^−1^ PFD was under G, at 100 μmol m^−2^ s^−1^ PFD—under R, and at 200 μmol m^−2^ s^−1^ PFD—under B. On the other hand, the presence of light could influence spore production. *C. gloeosporioides* sporulated intensely under 16 h of light [39]. In addition, *Podosphaera pannosa* conidia germination was reduced by blue (420 to 520 nm, peak 465 nm) light [34]. Additionally, blue (410–540 nm) light inhibits spore production of *P.* *digitatum* [30]. *C. acutatum* colony morphology differed under various PFD, and a 6 h photoperiod of blue (458 nm) LED light influenced its conidial germination and germ tube growth [36]. According to these results, light inhibits or reduces spore production. These findings could serve to pathogen ability to spread. Our results revealed that PFD and LED light influenced conidia size. The lowest width of conidia was under FR and length under G at 50 μmol m^−2^ s^−1^ PFD. The length of the conidia was lowest under FR and width under G at 100 μmol m^−2^ s^−1^ PFD. Besides this, the lowest conidia width was under G and length under R at 200 μmol m^−2^ s^−1^ PFD. Our results show differences in mycelial growth, conidia size, color, and recovery in different LEDs and PFD. In addition, various LED light wavelengths influenced *C. acutatum* colonies’ color. The results revealed that under W and B, mycelium was orange, under R and FR—light orange. Overall, *C. acutatum* colonies’ color varies among different LED light wavelengths, despite PFD. The recovery of *C. acutatum* under different LED light wavelengths and PFD revealed the slowest recovery observed under W at 100 μmol m^−2^ s^−1^ PFD. However, faster recovered under W at 200 μmol m^−2^ s^−1^ PFD. *C. acutatum* morphological and phenotypical characteristics transformations could be related to the ability of the fungus to spread and infect plant tissues.

Furthermore, as in control environment conditions, supplemental light is essential. Therefore, as light influences fungal pathogens, the selection of specific LEDs and PFD could help suppress and control strawberry fungal pathogen *C. acutatum.* LEDs in plant protection leads to optimal and appropriate technology usage. In addition, LEDs illumination can be combined with other disease control methods. The findings of this study will serve for future research.

## 4. Materials and Methods

The experiments were carried out at the Lithuanian Research Centre for Agriculture and Forestry (LAMMC IH), Institute of Horticulture, Laboratory of Plant Physiology under closed, controlled environment conditions in 2018–2019.

### 4.1. Fungal Isolate and Its Cultivation

Single spore *C. acutatum* F-05-I isolate was obtained from LAMMC IH Laboratory of plant protection isolate collection. The F-05-I origin was from the rotten strawberry fruit. The isolate was identified by PCR as *C. acutatum* by Xie et al. [40]. For experiments, isolates were cultivated in Petri plates containing potato dextrose agar (PDA) (Liofilchem, Roseto degli Abruzzi, Italy) at 25 ± 2 °C for 7 DAI. The isolates mycelial plugs (7 mm diameter) mycelium were placed side-down in the center of the new Petri with PDA. The morphological and phenotypic characteristics of *C. acutatum* were evaluated under different LEDs and PFD. The closed, controlled environment conditions were 23 ± 2 °C temperature and four hours (h) photoperiod. The control plates were incubated in complete darkness. There were four replicates per treatment.

### 4.2. Light Treatments and Intensity

For experiments, five different LEDs and three PFD treatments were selected. The light source was six LEDs wavelengths, as follows: B—blue (peak = 450 nm), G—green (peak = 530 nm), R—red (peak = 660 nm), FR—far red (peak = 735 nm), and W—white (5700 K) (Heliospectra RX30, Gothenburg, Sweden). The PFD was 50, 100 and 200 μmol m^−^^2^ s^−1^. The intensity of light was measured by photometer-radiometer RF-100 (Sonopan, Bialystok, Poland). The distance from the fungal sample to the light source was 40 cm.

### 4.3. Phenotypic and Morphological Characteristics Evaluation

*C. acutatum* was evaluated for phenotypic (conidia presence and size, mycelium growth rate) and morphological (mycelium appearance) characteristics. The mycelium growth rate was evaluated by measuring isolates diameter daily (mm day^−1^) and calculated as an average length and width increase per day for five days after inoculation (1–5 DAI).

The mycelium growth rate was used for mycelial growth inhibition (MGI) calculation [41].

$$\mathrm{MGI}\;\left(\%\right)=\frac{C-T}{C}\times 100,$$

where C—*C. acutatum* mycelium growth rate diameter in control, mm; T—*C. acutatum* mycelium growth rate diameter in treatment, mm.

Mycelial growth curve (AUMGC) was calculated by the formula [42]

$$\mathrm{AUMGC}=\sum ((y_{i}+y_{i}+1)/2\times \mathrm{dti})/n;$$

where *y_i_* = diameter of mycelium (mean) in the four observation, mm, dti = interval between evaluations, n = evaluation period.

The appearance of mycelium, presence of conidia, and size (width and length) were evaluated after 5 DAI. A conidia size (width and length, μm) and presence were evaluated in the plate’s margin with a Nikon Eclipse 80i microscope of 40× magnification after 5 DAI. The color of mycelium upper side at white and background evaluated 5 DAI. The mycelium color types were as follows: (1) white, (2) light orange, (3) orange. Three replications were maintained for each light and PFD treatment.

The re-isolation after 5 DAI was to evaluate the recovery of *C. acutatum* after treatment. The re-isolated growth rate (mm) measured after 48 h.

### 4.4. Statistical Analysis and Principal Component Analysis

The experimental data were analyzed with the software ANOVA from the program *SAS Enterprise Guide*, version 7.1 (SAS Inc., Cary, NC, USA). The standard error (SE) marked in the figures as an error bar was estimated for the growth rates of the isolates. Duncan’s Multiple Range Test used to determine differences among treatments. Conidia size and re-isolation data expressed as mean ± standard deviation.

The principal component analysis (PCA) was performed using Addinsoft XLSTAT 2019.1 XLSTAT statistical and data analysis solution (Long Island, NY, USA). The results presented in PCA Biplots indicate distinct effects of lighting treatments (B, G, R, FR, W) on *C. acutatum* mycelium growth at 1, 2, 3 and 4 days after infection (1 DAI, 2 DAI, 3 DAI, and 4 DAI, respectively) (based on Pearson’s correlation matrix).

## 5. Conclusions

Our results allowed exploring original research data techniques and leads to green plant protection solutions. Additionally, it raised new scientific questions for further research to develop environmentally safe plant protection methods of *C. acutatum* management in closed environmental conditions. The highest inhibition of *C. acutatum* achieved at 50 μmol m^−2^ s^−1^ PFD under G, at 100 μmol m^−2^ s^−1^ PFD—R, and 200 μmol m^−2^ s^−1^ PFD under B after 4 DAI. The lowest conidia width was under G and length under R. The *C. acutatum* mycelium color varied, and under G, R, and FR was light orange, and under B and W—orange color under different wavelengths at 5 DAI. The slowest recovery was at 50 μmol m^−2^ s^−1^ PFD under B, but at 100 μmol m^−2^ s^−1^ PFD—under W, and at 200 μmol m^−2^ s^−1^ PFD—under B. In conclusion, different PFD and LEDs influence *C. acutatum* growth characteristics. Furthermore, LEDs and PFD effects on *C. acutatum* growth characteristics are valuable for strawberries and other crops exposed to this pathogen. However, this research is in the initial stage, and further research is needed to develop innovative plant protection techniques for strawberry *C. acutatum* control.

## Figures and Tables

**Table 1:** Factor loadings, eigenvalues, variability (%), cumulative variability (%), and scores for the 50 (A), 100 (B) and 200 (C) µ mol m^−2^ s^−1^ photon flux density for *C. acutatum* mycelium growth measurements under five different LED light wavelengths on first-four days after inoculation. The PCA Biplot A, B, C indicates treatments of 50, 100, and 200 µ mol m^−2^ s^−1^ photon flux density, respectively. DAI—days after inoculation; B—blue (peak = 450 nm); G—green (peak = 530 nm); R—red (peak = 660 nm); FR—far red (peak = 735 nm); W—white (5700 K).

PCA Biplot	A		B		C	
Factors	F1	F2	F1	F2	F1	F2
Eigenvalue	2.042	1.041	2.225	1.044	3.091	0.513
Variability (%)	51.047	26.013	55.626	26.110	77.265	12.836
Cumulative variability (%)	77.060		81.736		90.100	
Factor loadings						
1 DAI	0.651	−0.614	0.888	−0.136	0.779	−0.622
2 DAI	0.786	0.282	0.833	0.289	0.912	0.315
3 DAI	0.517	0.728	−0.194	0.967	0.920	0.064
4 DAI	0.856	−0.232	0.839	0.081	0.897	0.154
Factor scores						
B	−0.5736	0.8253	0.951	1.096	−2.3484	0.0937
G	−1.4932	0.5292	1.678	−1.644	−0.3940	−0.4173
R	1.2799	−0.3262	−1.331	0.052	0.6982	0.5869
FR	0.5885	−1.1771	−1.236	0.047	1.2974	−0.1943
W	0.1984	0.1488	−0.061	0.449	0.7469	−0.0690

**Table 2:** The mycelial growth curve (AUMGC) of *C. acutatum* under different photosynthetic photon flux densities.

LED Light Wavelengths	Photosynthetic Photon Flux Density		
	50 µ mol m^−2^ s^−1^	100 µ mol m^−2^ s^−1^	200 µ mol m^−2^ s^−1^
B	10.22 ± 0.41	11.33 ± 0.33	9.37 ± 0.24
G	9.94 ± 0.21	11.58 ± 0.29	9.99 ± 0.38
R	10.94 ± 0.32	10.30 ± 0.23	10.37 ± 0.27
FR	10.53 ± 0.48	10.36 ± 0.33	10.58 ± 0.34
W	10.46 ± 0.29	10.71 ± 0.36	10.39 ± 0.28

Results are presented as mean ± SD (*n* = 4) (*p* < 0.05). LED light wavelengths: B—blue (peak = 450 nm); G—green (peak = 530 nm); R—red (peak = 660 nm); FR—far red (peak = 735 nm); W—white (5700 K).

**Table 3:** Comparison of the average conidia size of *C. acutatum* after illumination by various LED light photosynthetic photon flux densities.

Conidia Size, μm	LED Light Wavelengths				
	B	G	R	FR	W
	50 µ mol m^−2^ s^−1^ photosynthetic photon flux density				
Width	9.0 ± 0.6	8.1 ± 0.5	9.1 ± 0.3	7.6 ± 0.3	10.3 ± 0.5
Length	26.5 ± 3.0	23.8 ± 1.8	27.5 ± 2.5	23.9 ± 1.8	33.3 ± 2.7
	100 µ mol m^−2^ s^−1^ photosynthetic photon flux density				
Width	8.9 ± 0.5	8.6 ± 0.6	9.2 ± 0.7	9.6 ± 0.4	9.9 ± 0.5
Length	24.7 ± 1.0	23.3 ± 1.2	26.8 ± 2.0	21.5 ± 2.4	30.1 ± 2.1
	200 µ mol m^−2^ s^−1^ photosynthetic photon flux density				
Width	9.4 ± 0.6	8.3 ± 0.7	8.7 ± 0.3	9.1 ± 0.5	9.3 ± 0.6
Length	26.2 ± 1.9	25.0 ± 0.8	24.9 ± 1.9	30.5 ± 0.6	30.0 ± 0.9

Results are presented as mean ± SD (*n* = 4) (*p* < 0.05). LED light wavelengths: B—blue (peak = 450 nm); G—green (peak = 530 nm); R—red (peak = 660 nm); FR—far red (peak = 735 nm); W—white (5700 K).

**Table 4:** The average recovery of *C. acutatum* mycelium after illumination by various LED light photosynthetic photon flux densities, mm.

LED Light Wavelengths				
B	G	R	FR	W
50 µ mol m^−2^ s^−1^ photosynthetic photon flux density				
25.8 ± 0.3 a	26.5 ± 0.5 a	28.9 ± 0.2 d	28.4 ± 0.1 cd	27.8 ± 0.2 bcd
100 µ mol m^−2^ s^−1^ photosynthetic photon flux density				
24.5 ± 0.7 abc	26.0 ± 0.4 c	23.8 ± 0.1 a	24.8 ± 0.1 abc	23.6 ± 0.2 a
200 µ mol m^−2^ s^−1^ photosynthetic photon flux density				
24.1 ± 0.2 b	24.5 ± 0.2 b	24.6 ± 0.1 b	24.4 ± 0.1 b	27.4 ± 0.6 c

Results are presented as mean ± SD (*n* = 4) (*p* < 0.05). According to Duncan’s multiple range test, the means of different letters are significantly different at the *p* < 0.05 level. LED light wavelengths: B—blue (peak = 450 nm); G—green (peak = 530 nm); R—red (peak = 660 nm); FR—far red (peak = 735 nm); W—white (5700 K).

## Data Availability

All data included in the main text.

## Supplementary Materials

The following supporting information can be downloaded at: www.mdpi.com/article/10.3390/plants11010143/s1, Table S1: Correlation matrix (Pearson (n)) at 50 µ mol m^−2^ s^−1^; Table S2: Correlation matrix (Pearson (n)) at 100 µ mol m^−2^ s^−1^; Table S3: Correlation matrix (Pearson (n)) at 200 µ mol m^−2^ s^−1^.

## References

[1] Fung F., Wang H.S., Menon S. (2018). Food safety in the 21st century. Biomed. J..

[2] Rather I.A., Koh W.Y., Paek W.K., Lim J. (2017). The Sources of chemical contaminants in food and their health implications. Front. Pharmacol..

[3] Tiilikkala K., Lindqvist I., Hagner M., Setälä H., Perdikis D., Stoytcheva M. (2011). Use of botanical pesticides in modern plant protection. Pesticides in the Modern World—Pesticides Use and Management.

[4] Stentiford G.D., Becnel J., Weiss L.M., Keeling P.J., Didier E.S., Williams B.P., Bjornson S., Kent M.L., Freeman M.A., Brown M.J.F. (2016). Microsporidia—Emergent Pathogens in the Global Food Chain. Trends Parasitol..

[5] Elad Y., Vivier M., Fillinger S., Fillinger S., Elad Y. (2016). *Botrytis*, the good, the bad and the ugly. Botrytis—The Fungus, the Pathogen and Its Management in Agricultural Systems.

[6] Carisse O., Morissette-Thomas V., Van der Heyden H. (2013). Lagged association between powdery mildew leaf severity, airborne inoculum, weather, and crop losses in strawberry. Phytopathology.

[7] Elad Y., Fillinger S., EditorElad Y. (2016). Cultural and integrated control of *Botrytis*. Botrytis—The Fungus, the Pathogen and Its Management in Agricultural Systems.

[8] Kim K., Kook H.-S., Jang Y.-J., Lee W.-H., Kamala-Kannan S., Chae J.-C., Lee K.-J. (2013). The effect of blue-light emitting diodes on antioxidant properties and resistance to *Botrytis cinerea* in tomato. J. Plant Pathol. Microbiol..

[9] Kook H.S., Park S.H., Jang Y.J., Lee G.W., Kim J.S., Kim H.M., Oh B.T., Chae J.C., Lee K.J. (2013). Blue LED (light-emitting diodes)-mediated growth promotion and control of *Botrytis disease* in lettuce. Acta Agric. Scand. B Soil Plant Sci..

[10] Damos P., Colomar L.A., Ioriatti C. (2015). Integrated fruit production and pest management in Europe: The apple case study and how far are from the original concept?. Insects.

[11] Beckerman J.L., Sundin G.W., Rosenberger D.A. (2015). Do some IPM concepts contribute to the development of fungicide resistance? Lessons learned from the apple scab pathosystem in the United States. Pest Manag. Sci..

[12] Morkeliūnė A., Rasiukevičiūtė N., Valiuškaitė A. (2021). Pathogenicity of *Colletotrichum acutatum* to different strawberry cultivars and anthracnose control with essential oils. Zemdirb.-Agric..

[13] D’Souza C., Yuk H., Khoo H.G., Zhou W. (2015). Application of light-emitting diodes in food production, postharvest preservation, and microbiological food safety. Compr. Rev. Food Sci. Food Saf..

[14] Hasan M.M., Bashir T., Ghosh R., Lee S.K., Bae H. (2017). An Overview of LEDs’ effects on the production of bioactive compounds and crop quality. Molecules.

[15] Šernaitė L., Valiuškaitė A., Rasiukevičiūtė N., Dambrauskienė E., Viškelis P. (2020). Effectiveness of mixtures and individual plant extracts and essential oils for biocontrol of *Botrytis cinerea*. Žemdirb.-Agric..

[16] Freeman S., Horowitz S., Sharon A. (2001). Pathogenic and nonpathogenic life style in *Colletotrichum acutatum* from strawberry and other plants. Phytopathology.

[17] Aguado A., Pastrana A.M., Santos B., Romero F., Sánchez M.C., Capote N. (2014). The efficiency of natural products for the control of *Colletotrichum acutatum* monitored by real-time PCR. Acta Hort..

[18] Feliziani E., Romanazzi G. (2016). Postharvest decay of strawberry fruit: Etiology, epidemiology, and disease management. J. Berry Res..

[19] Cannon P.F., Damm U., Johnston P.R., Weir B.S. (2012). *Colletotrichum*—Current status and future directions. Stud. Mycol..

[20] Udayanga D., Manamgoda D.S., Liu X., Chukeatirote E., Hyde K.D. (2013). What are the common anthracnose pathogens of tropical fruits?. Fungal Divers..

[21] Sreenivasaprasad S., Talhinhas P. (2005). Genotypic and phenotypic diversity in *Colletotrichum acutatum*, a cosmopolitan pathogen causing anthracnose on a wide range of hosts. Mol. Plant Pathol..

[22] Bian Z.H., Yang Q.C., Liu W.K. (2015). Effects of light quality on the accumulation of phytochemicals in vegetables produced in controlled environments: A review. J. Sci. Food Agric..

[23] Vaštakaitė V., Viršilė A., Brazaitytė A., Samuolienė G., Jankauskienė J., Novičkovas A., Duchovskis P. (2017). Pulsed Light-Emitting diodes for a higher phytochemical level in microgreens. J. Agric. Food Chem..

[24] Brazaitytė A., Viršilė A., Samuolienė G., Vaštakaitė-Kairienė V., Jankauskienė J., Miliauskienė J., Novičkovas A., Duchovskis P. (2019). Response of mustard microgreens to different wavelengths and durations of UV-A LEDs. Front. Plant Sci..

[25] Bantis F., Smirnakou S., Ouzounis T., Koukounaras A., Ntagkas N., Radoglou K. (2018). Current status and recent achievements in the field of horticulture with the use of light-emitting diodes (LEDs). Sci. Hortic..

[26] Paul N.D., Rasanayagam S., Moody S.A., Hatcher P.E., Ayres P.G. (1997). The role of interactions between trophic levels in determining the effects of UV-B on terrestrial ecosystems. Plant Ecol..

[27] Schumacher J. (2017). How light affects the life of *Botrytis*. Fungal Genet. Biol..

[28] Schumacher J., Gorbushina A.A. (2020). Light sensing in plant- and rock-associated black fungi. Fungal Biol..

[29] Costa T.P.C., Rodrigues E.M., Dias P.D., Pupin B., Ferreira P.C., Rangel D.E.N. (2021). Different wavelengths of visible light influence the conidial production and tolerance to ultra-violet radiation of the plant pathogens *Colletotrichum acutatum* and *Fusarium fujikuroi*. Eur. J. Plant Pathol..

[30] Liao H.-L., Alferez F., Burns J.K. (2013). Assessment of blue light treatments on citrus postharvest diseases. Postharvest Biol. Technol..

[31] Ballaré C.L. (2014). Light Regulation of Plant Defense. Annu. Rev. Plant Biol..

[32] Alferez F., Liao H.-L., Burns J.K. (2012). Blue light alters infection by *Penicillium digitatum* in tangerines. Postharvest Biol. Technol..

[33] Xu H., Fu Y., Li T., Wang R. (2017). Effects of different LED light wavelengths on the resistance of tomato against *Botrytis cinerea* and the corresponding physiological mechanisms. J. Integr. Agric..

[34] Suthaparan A., Torre S., Stensvand A., Herrero M.L., Pettersen R.I., Gadoury D.M., Gislerød H.R. (2010). Specific light-emitting diodes can suppress sporulation of *Podosphaera pannosa* on greenhouse roses. Plant Dis..

[35] Alsanius B.W., Karlsson M., Rosberg A.K., Dorais M., Naznin M.T., Khalil S., Bergstrand K.-J. (2019). Light and microbial lifestyle: The impact of light quality on plant–microbe interactions in horticultural production systems—A review. Horticulturae.

[36] Yu S.-M., Ramkumar G., Lee Y.H. (2014). Light quality influences the virulence and physiological responses of *Colletotrichum acutatum* causing anthracnose in pepper plants. J. Appl. Microbiol..

[37] Kommula S.K., Reddy G.P.D., Undrajavarapu P., Kanchana K.S. (2017). Effect of Various Factors (Temperature, pH and Light Intensity) on Growth of *Colletotrichum capsici* Isolated from Infected Chilli. Int. J. Pure App. Biosci..

[38] Simkovitch R., Gajst O., Zelinger E., Yarden O., Huppert D. (2017). Irradiation by blue light in the presence of a photoacid confers changes to colony morphology of the plant pathogen *Colletotrichum gloeosporioides*. J. Photochem. Photobiol. B.

[39] Yoon J.B., Park H.G. (2001). Screening method for resistance to pepper fruit anthracnose: Pathogen sporulation, inoculation methods related to inoculum concentrations and post-inoculation environment. Korean J. Hortic. Sci. Technol..

[40] Xie L., Zhang J., Wan Y., Hu D. (2010). Identification of *Colletotrichum* spp. isolated from strawberry in Zhejiang Province and Shanghai City, China. J. Zhejiang Univ. Sci. B.

[41] Cherkupally R., Kota R., Amballa H., Reddy B.N. (2017). *In vitro* antifungal potential of plant extracts against *Fusarium oxysporum, Rhizoctonia solani* and *Macrophomina phaseolina*. Ann. Plant Sci..

[42] Campbell C.L., Madden L.V. (1990). Introduction to Plant Disease Epidemiology.

