# Supplementary material for: Different LED Light Wavelengths and Photosynthetic Photon Flux Density Effect on Colletotrichum acutatum Growth
Source: Plants (Basel). 2022 Jan 5;11(1):143. doi: 10.3390/plants11010143 (PMC8747561; doi:10.3390/plants11010143)
Supplement: Supplementary file 1 [file plants-11-00143-s001.zip › plants-1501955-supplementary.pdf]

plants

MDPI

Table S1. Correlation matrix (Pearson (n)) at 50 μ mol m^{-2} s^{-1}

Variables	1 DAI	2 DAI	3 DAI	4 DAI
1 DAI	**1**	0.246	0.043	**0.541**
2 DAI	0.246	**1**	**0.392**	**0.532**
3 DAI	0.043	**0.392**	**1**	0.237
4 DAI	**0.541**	**0.532**	0.237	1

Values in bold are different from 0 with a significance level alpha=0.05

Table S2. Correlation matrix (Pearson (n)) at 100 μ mol m^{-2} s^{-1}

Variables	1 DAI	2 DAI	3 DAI	4 DAI
1 DAI	**1**	**0.630**	-0.246	**0.614**
2 DAI	**0.630**	**1**	0.055	**0.561**
3 DAI	-0.246	0.055	**1**	-0.078
4 DAI	**0.614**	**0.561**	-0.078	**1**

Values in bold are different from 0 with a significance level alpha=0.05

Table S3. Correlation matrix (Pearson (n)) at 200 μ mol m^{-2} s^{-1}

Variables	1 DAI	2 DAI	3 DAI	4 DAI
1 DAI	**1**	**0.532**	**0.653**	**0.604**
2 DAI	**0.532**	**1**	**0.835**	**0.807**
3 DAI	**0.653**	**0.835**	**1**	**0.728**
4 DAI	**0.604**	**0.807**	**0.728**	**1**

Values in bold are different from 0 with a significance level alpha=0.05
